# Trial by Fire: Experiences of the 2020 Graduates of Pediatric Hospital Medicine Fellowships

**DOI:** 10.7759/cureus.93092

**Published:** 2025-09-24

**Authors:** Jan Fune, Jeri Kessenich, Weixin Li, Bian Liu, Leora Mogilner

**Affiliations:** 1 Pediatrics, Icahn School of Medicine at Mount Sinai, New York, USA; 2 Pediatrics, Michigan State University College of Human Medicine, Grand Rapids, USA; 3 Population Health Science and Policy, Icahn School of Medicine at Mount Sinai, New York, USA

**Keywords:** faculty satisfaction, fellowship curriculum, fellowship education, fellowship training, graduate medical education (gme), level of job satisfaction, pediatric hospital medicine, physician satisfaction, preparedness training, transition-to-practice

## Abstract

Background and objective

There is scarce data regarding the preparedness of pediatric hospital medicine (PHM) fellowship graduates for their first attending physician position. Hence, we sought to investigate how prepared PHM fellowship graduates felt in terms of attending-level skills and their satisfaction with their first job.

Methods

We conducted a national cross-sectional survey with open- and closed-ended questions to assess preparedness for topics taught in the PHM fellowship, and included questions to assess job satisfaction. In 2021, a survey was sent to PHM attendings who graduated from a PHM fellowship in 2020 (approximately one year into their first job). Participants were asked to rate their preparedness for 25 different topics on a 5-point Likert scale and to rate their satisfaction with their fellowship, fellowship director, and first job. We used descriptive statistics for the closed-ended questions. Answers to the open-ended questions were coded on the keywords of each answer.

Results

Twenty-one out of 42 attendings completed the survey, yielding a 50% response rate. Respondents felt most prepared to lead rounds (4.81/5, standard deviation [SD]: 0.60), teach residents (4.62/5, SD: 0.50), teach medical students (4.43/5, SD: 0.75), manage complex patients (4.19/5, SD: 0.87), and perform consults (4/5, SD: 1.26), and felt least prepared in neonatal resuscitation (2.24/5, SD: 1.41), sedation (1.86/5, SD: 1.01), and point-of-care ultrasound (1.57/5, SD: 0.98). On average, participants were moderately satisfied with their first job after the fellowship (average rating score: 4.33, range: 3-5). Respondents described areas of improvement for fellowship education, including preparation for their first attending job.

Conclusions

Our study reveals the areas in which respondents felt most and least prepared to perform as a new attending physician. Our findings can assist PHM fellowship program directors in preparing their trainees for entry into the workforce.

## Introduction

The transition from trainee to attending physician can be a stressful experience. Preparedness for tasks such as practicing independent patient care and performing administrative and educational tasks, as well as adapting to a new work environment, can lead to poor job satisfaction [1]. To address these issues, the Accreditation Council for Graduate Medical Education (ACGME) establishes educational and professional standards, as well as curricular components, that are essential in preparing physicians for autonomous practice [2]. However, there is a paucity of research investigating post-graduate job satisfaction or assessing the preparedness of pediatric and adult medicine trainees for independent practice in the literature [3-6]. In a study by Mery et al., researchers identified the transition from congenital heart surgery fellow to clinical practice as a problem, with 91% not feeling ready to engage in solo practice after completing ACGME-accredited fellowship training [4]. In another study, researchers gathered data on operative experience, job transition, and job satisfaction of congenital heart surgery fellowship graduates from ACGME-accredited programs. They found the operative experience improved after accreditation, but only 44% felt strongly that the fellowship prepared them for independent practice [5].

The difficulty in transitioning to attending status is highlighted in several studies. A 2021 study by Weiss et al. found that the majority of fellowship directors of ACGME-certified pediatric fellowships did not require graduating fellows to be trusted or prepared to practice without supervision or coaching in any of the entrustable professional activities (EPAs) that drive fellowship training [7]. This was especially true for the non-clinical work of new attending subspecialists (e.g., billing, team leadership, administrative tasks, quality improvement, and research). The respondents in this study completed the survey in 2017, before Pediatric Hospital Medicine (PHM) became an ACGME-accredited fellowship, and it was published before many PHM fellowships existed.

A 2014 survey study found that PHM fellowship graduates identified areas of ongoing high perceived needs, such as procedures, hospital program management, QI methodology, and curriculum development [8]. Lastly, a study by Rassbach et al., which surveyed PHM graduates from 2000 to 2018, found that graduates of two- and three-year fellowships were significantly more productive than those who only completed one-year programs. Their qualitative data analysis showed that fellowships prepare graduates well for diverse academic career paths [9]. However, they also hypothesized that the shift toward two-year programs would lead to changes in clinical time and scholarly productivity.

To assess if PHM fellowship graduates during the time of ACGME accreditation felt prepared for their first attending job, we surveyed pediatric hospitalists who graduated from a PHM fellowship in 2020. Our primary aims were to assess how prepared PHM fellowship graduates felt in performing attending-level skills in the domains of clinical, administrative, scholarship, and career advancement, and how satisfied they were with their first job as an attending. As for our secondary aims, we explored the factors that contributed to their selection of their first job, any barriers they faced during their job search, and the surprises they discovered during their first year as an attending. Additionally, we explored how they felt their fellowship program could have better prepared them for their first job.

## Materials and methods

Study design

We conducted a cross-sectional survey of PHM attendings who graduated from a PHM fellowship in 2020. Participants were included if they had graduated from a two- or three-year PHM fellowship program and excluded if they had graduated from a one-year program, or any year before 2020. Surveys were emailed in August 2021, meaning that respondents would have been in their first job for approximately one year. This study was deemed exempt by the Icahn School of Medicine Institutional Review Board (IRB), the lead author’s IRB at the time the research was conducted.

Survey development

The survey (Appendix 1) was prepared by a PHM fellowship program director, a recent PHM fellowship graduate, and an epidemiologist, with additional review performed by an external PHM fellowship program director. Items, such as patient care and procedural skills (e.g., provide consultation, manage complex care patients, and perform lumbar puncture) were selected from the 2020 ACGME requirements for graduate medical education in PHM [10-11]. To ensure content and response process validity, we piloted the survey with PHM fellowship graduates who completed their fellowship before 2020. Questions were added, modified, or removed based on feedback from pilot survey users, and the survey was modified until we were satisfied; their responses were excluded from the final data analysis. We used a combination of closed- and open-ended questions to assess job preparedness and satisfaction.

Survey instrument

Close-ended questions assessed or pertained to the following topics: (1) demographics and fellowship program characteristics, (2) preparedness for 25 different aspects of a hospitalist job on a 5-point Likert scale (extremely prepared to not at all prepared), (3) satisfaction with the fellowship program on a 5-point Likert scale (extremely satisfied to not at all satisfied), (4) satisfaction with their fellowship program director on a 5-point Likert scale (extremely satisfied to not at all satisfied), (5) how helpful was the fellowship director in finding the first job (extremely helpful to not at all helpful), (6) how confident are you that fellowship prepared you for your first job (extremely confident to not at all confident), (7) how satisfied are you with your first job after fellowship (extremely satisfied to not at all satisfied), (8) have you met with a mentor at your first job, and (9) did your fellowship offer a career development curriculum. Close-ended questions about “preparedness” were selected based on the 2020 ACGME program requirements for graduate medical education in PHM [10-11].

Open-ended, free-text response questions addressed the respondents’ first job, including surprises, challenges, and barriers encountered, as well as ways in which the fellowship could have better prepared the respondents for attending practice. Participants were given 18 factors and asked to identify and rank the five most important factors they considered when choosing the first job (e.g., location, salary, size of the division).

Data collection

We generated a list of all the PHM fellows who graduated in 2020. Specifically, we obtained the majority of emails from a directory created during the annual Fellows Conference sponsored by the American Academy of Pediatrics Section on Hospital Medicine in 2018 and 2019. We contacted individual PHM fellowship program directors to obtain the emails of the fellows who did not attend the conference in those years. The survey (Appendix 1) was emailed to 42 PHM fellowship graduates in August 2021, presumably one year into participants’ first year in their first attending job. Two subsequent email reminders were sent one month apart. There were no incentives to participate in the survey.

Statistical analysis

We used descriptive statistics for the closed-ended questions. Frequency and proportion were calculated to summarize categorical variables. For questions with Likert-scale responses, we summarized the average score using both the mean and standard deviation (SD) as well as the median and interquartile range (IQR) to better capture the distribution of responses. Missing inputs were minimal and are described in the table captions. The analysis was conducted using SAS (version 9.4TS1m6). Answers to the open-ended questions were independently reviewed by three faculty members who developed initial codes and then met to reach consensus on final categories based on the keywords of each response.

## Results

Demographics of the participants

A total of 21 participants completed the survey out of 42 invited, yielding a response rate of 50%. Except for one participant who attended a three-year fellowship, all the other participants attended two-year fellowships. All 21 participants graduated from their fellowship program in 2020 (Table 1). The majority of the participants were females (n=17; Table 1). Among the participants, 13 self-identified as White or European American, four were Asian, two were Hispanic, and one was Black or African American. Most of the participants were between the ages of 31 and 35 years (n=18).

**Table 1:** Demographics and fellowship program characteristics of the 21 participants This table summarizes the demographic and training-related characteristics of PHM fellowship graduates who responded to the survey. Data were collected using structured, closed-ended questions and are reported as frequencies and percentages PHM: pediatric hospital medicine

Demographics and fellowship program characteristics		
Demographics	Frequency	Percent
Age group, years		
25-30	1	4.8
31-35	18	85.7
36-40	2	9.5
Gender		
Male	4	19.1
Female	17	80.9
Race		
White or European American	13	61.8
Asian	7	19.1
East Asian	2	
East Asian and Asian-American	1	
South Asian	1	
Hispanic	2	9.5
Black or African American	1	4.8
Prefer not to say	1	4.8
Other	0	0
Fellowship program characteristics	Frequency	Percent
Graduation year		
2020	21	100
Length of fellowship, years		
2	20	95.2
3	1	4.8
Career development curriculum		
Yes	10	47.6
Fellowship program level	2	
Departmental/institutional level	2	
Both levels	6	
No	5	23.8
Unsure	6	28.6

Preparedness

On average, participants felt that their fellowship prepared them at least moderately well in 20 out of 25 topics (average rating score ≥3) covered during their fellowship (Table 2). The participants were most satisfied with the following five topics (average rating score ≥4): leading family-centered rounds, teaching residents, teaching medical students, managing complex care patients, and performing hospital medicine consults. Respondents felt less prepared (average rating score <3) in the subjects of billing, the promotion process, neonatal resuscitation, sedation, and point-of-care ultrasound. 

**Table 2:** Average rating scores of the 25 topics covered during the fellowship program for attending preparedness This table displays the mean Likert-scale ratings for 25 core training topics covered during the PHM fellowship, assessing how well each topic prepared graduates for their attending role. Ratings range from 1 (not at all prepared) to 5 (extremely prepared). One respondent did not rate "hospital medicine consults", resulting in a single missing value for that item. Topics were selected based on ACGME training requirements SD: standard deviation; IQR: interquartile range; PHM: pediatric hospital medicine; ACGME: Accreditation Council for Graduate Medical Education

Topic	Average rating on fellowship preparedness topics	SD	Median (IQR)
Leading family-centered rounds	4.81	0.60	5 (5–5)
Teaching residents	4.62	0.50	5 (4–5)
Teaching medical students	4.43	0.75	5 (4–5)
Managing complex care patients	4.19	0.87	4 (4–5)
Hospital medicine consults	4.00	1.26	4 (3.75–5)
Quality improvement	3.95	0.74	4 (3–4)
Mentoring residents	3.86	1.06	4 (3–5)
Mentoring medical students	3.71	1.23	4 (3–5)
Publishing scholarly work	3.67	0.97	4 (3–4)
Pain management	3.62	0.86	4 (3–5)
Work with non-physician providers	3.62	1.24	3 (3–4)
Finding a job after the fellowship	3.57	1.12	4 (3–4)
Lumbar puncture	3.48	1.17	3 (3–4)
Clinical research	3.43	1.16	4 (3–4)
Maintaining wellness	3.43	0.98	3 (3–4)
Surgical co-management	3.29	1.19	3 (2–4)
Navigating hospital politics	3.24	0.83	3 (3–4)
Operational leadership	3.14	1.31	3 (2–4)
Medical education research	3.10	1.22	3 (2–4)
Pediatric resuscitation and stabilization	3.05	0.92	3 (2–4)
Billing	2.86	1.31	3 (2–4)
Promotion process	2.24	0.94	2 (1–3)
Neonatal resuscitation	2.24	1.41	2 (2–3)
Sedation	1.86	1.01	2 (1–2)
Point-of-care ultrasound	1.57	0.98	1 (1–2)

The first job: factors contributing to selection, satisfaction, challenges in transitioning to a new role, and surprises

**Figure 1:**
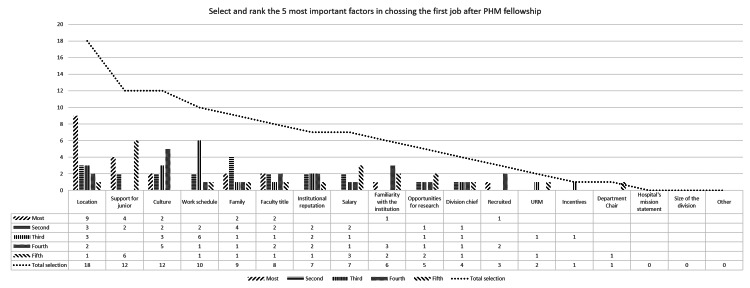
Selection and ranking of the 5 most important factors in choosing the first job after PHM fellowship This figure illustrates the frequency with which each of 18 job selection factors was ranked among the top five by PHM fellowship graduates when choosing their first job post-fellowship. Respondents selected and ranked the five most important factors from a pre-defined list, including items. Factors are ordered by frequency of selection PHM: pediatric hospital medicine

Figure 1 summarizes the frequency of each factor that participants selected and ranked when choosing their first job. The most frequently mentioned factors that were important in choosing the first job after the PHM fellowship were location (n=18, 85.7%), support for junior faculty (n=12, 57.1%), culture of the hospitalist division (n=12, 57.1%), work schedule (n=10, 47.6%), and family (n=9, 42.9%). The factors that most frequently ranked as the most important factor were location (n=9), support for junior faculty (n=4), culture (n=2), family (n=2), and faculty title (n=2). On average, participants were moderately satisfied with their first job after fellowship (average rating score 3.67 (SD: 1.32), range 1-5).

When asked “what surprised you the most about your first job?”, the most common surprise at the first job was how different it was compared to the fellowship (n=6) and the difficulty finding mentorship (n=5) (Table S6 in the Appendix). Three participants found it surprisingly difficult to adapt to the new environment, while three found the transition surprisingly smooth. When asked to elaborate on why they were surprised, three participants responded that it was due to the difference in institutional culture between the fellowship and their first job. Two participants answered that they were surprised because there was less working time during the fellowship than in their first job. Other reasons included taking the mentorship for granted during the fellowship, expecting less workload at the first job, and receiving a different message than the reality of the job (Table S7 in the Appendix).

Regarding the most significant challenge transitioning from fellow to attending, the most frequently mentioned challenges were increased clinical responsibility and less support or supervision as an attending (n=11) (Table S8). Participants also found it challenging to adapt to their increased workload, learn and adapt to a new system, initiate scholarly work, transition from fellow to attending status when remaining at the same institution, master billing, establish clinical relationships, and find mentorship. Twelve out of 21 participants had identified a mentor at their first job, while eight participants had not identified a mentor.

Perceived barriers to finding the first job

Nearly half of the participants (n=10) mentioned the lack of job openings on the market as a barrier to finding the right job after fellowship, specifically during the pandemic (Table S9 in the Appendix). Five participants mentioned that balancing various factors in choosing a job was a barrier. Other identified barriers included finding a competitive salary and logistical difficulties navigating interviews and offers. Other perceived barriers mentioned included a lack of preparedness for the job search after the fellowship and being unable to visit and learn more before onboarding. Three participants reported that they did not encounter any barriers to finding the right job after completing their fellowship, while three respondents thought that completing the fellowship would increase their job opportunities beyond what they actually experienced.

Satisfaction with the fellowship program

Overall, participants were quite satisfied with the fellowship program (average rating score 4.33, range 3-5) and their primary fellowship director (average rating score 4.52 (SD: 0.75)). The participants were quite confident (mean score = 4.05 (SD 0.67)) that their fellowship program would prepare them for their first job. Additionally, except for one participant, all the other participants reported that the fellowship director was at least moderately helpful in finding their first job (average rating score: 4.14).

What fellowships can focus on to help graduates prepare for their first job?

Six participants mentioned that they would have benefited more from the fellowship program if they had received more training focusing on job preparation (e.g., job search, promotion process, salary) (Table 3). Four participants mentioned that they would prefer more training focusing on procedures and clinical skills that were less emphasized during residency (e.g., sedation, point-of-care ultrasound, lumbar puncture). Four participants mentioned they would have benefited from more training focusing on billing. Other identified areas for improvement included mentoring students and residents, responding to rapid responses, stress management, providing feedback to learners, promoting autonomy, medical leadership, and evidence-based medicine.

**Table 3:** The counts of what participants think would be beneficial if the fellowship focused on more This table shows the number of respondents who reported that additional emphasis on specific topics would have better prepared them for their first attending role. Responses were coded from free-text answers provided in open-ended survey questions POCUS: point-of-care ultrasound; LP: lumbar puncture

Key information	Exact words from the answers	Counts
Preparation for a job (e.g., job search, promotion process, salary)	“Working on my CV”	6
	“How to navigate promotion.”	
	“The job search and the promotion process”	
	“Preparing for job search…”	
	“1. Ins and outs of salaries/compensation… 2. How to navigate tricky professional situations”	
	“Learning how to negotiate and shape the job into what is desired”	
Procedures/clinical skills	“POCUS”	4
	“Neonatal resuscitation and stabilization”	
	“LP skills”	
	“Time on topics such as sedation, point of care ultrasound, other procedures/clinical skills that are not necessarily taught during residency…”	
Billing	“Devin billing and doing notes for purposes of billing…”	4
	“Billing - I feel as if there are very few people in the clinical realm that truly know how to bill…”	
	“Billing”	
	“Billing…”	
Mentoring students and residents	“Mentoring students	2
	“…Guidance on how to mentor residents and students effectively”	
Responsiveness	“Running rapid responses”	1
Stress management	“Stress management”	1
More feedback	“…I would have enjoyed more training on giving constructive feedback to learners.”	1
Autonomy	“More autonomy for resident teams”	1
Medical leadership	“Medical leadership”	1
Evidence-based medicine	“Evidence-based medicine/critically appraising articles.”	1

Ten participants reported that their fellowship program offered a career development curriculum; two were at the fellowship program level, two were at the departmental/institutional level, and six were at both levels. Six participants (28.6%) were unsure whether the fellowship program offered a career development curriculum, and five participants reported that the fellowship program did not offer a career development curriculum.

## Discussion

PHM fellowships offer fellows the opportunity to learn and practice the skills necessary for independent practice as a hospitalist. However, until recently, PHM fellowships and curricula were highly individualized as PHM was only granted designation as a subspecialty by the American Board of Medical Specialties in 2016, and the ACGME first published program requirements in 2019 [11]. Therefore, this cross-sectional study about the preparedness and satisfaction of PHM fellowship graduates from 2020 captures participants at a transitional phase and explores their experiences in fellowship and in their new jobs at this unique time point.

Similar to findings in prior studies [3-6,8], PHM fellowship graduates often graduate without feeling completely competent or prepared for their transition to attending. Our findings identify domains in which fellowship graduates felt most and least prepared to perform as new hospitalist attendings. Fellowship graduates felt most prepared for the tasks commonly emphasized during fellowship, including leading family-centered rounds, teaching residents and medical students, caring for complex care patients, and acting as a consultant to other services and subspecialties. This finding is generally in agreement with the work by Weiss et al., whereby fellowship program directors noted that the least amount of supervision needed for all pediatric fellowship graduates was in the areas of consultation and handovers, skills that PHM fellows have significant experience with during training [7].

Overall, our respondents felt prepared to publish scholarly work and perform research. This finding contrasts with a survey that concluded in 2023, which showed that current PHM fellows and recent graduates felt least prepared in this domain [12]. However, our findings revealed that new hospitalists feel much less prepared for tasks, including core competencies such as performing neonatal resuscitation, sedation, and point-of-care ultrasound, which respondents identified as areas that their PHM fellowship could have focused more on. These findings are consistent with prior studies, which found that PHM fellowship graduates felt they received adequate training in several domains except for procedural training, such as sedation [8,13].

Outside of core competencies, respondents felt least prepared in the subjects of billing, navigating the promotion process, and searching for a job. These findings are similar to those of Weiss et al., who noted that the highest level of supervision required for graduating PHM fellows was in the areas of workplace management, leading in the profession, scholarship, and quality improvement [7]. Free text responses revealed that graduates wanted more focused training on billing, which attending physicians are responsible for. Prior studies have shown that feeling unprepared is associated with poorer job satisfaction [1].

Despite feeling unprepared in five domains, participants in our study were moderately satisfied with their first job after completing the fellowship. The first PHM fellowships were only recently accredited by the ACGME in 2020, so this study sheds light on the actual experiences of fellows and the training they received during this time of transition to ACGME certification. It is possible that graduates felt less prepared to perform certain core competencies due to a lack of opportunities to practice these skills to the point of competency, or because they were not yet curricular components of the fellowship at that time. For example, the low number of opportunities for non-intensivists to practice sedation has been established in the literature. The Pediatric Sedation Research Consortium found that the majority of sedation cases using Propofol were done by intensivists and emergency medicine physicians, while only 2% of the cases were done by general pediatricians or hospitalists [14].

A narrative review of pediatric emergency medicine fellowship programs identified disparities in hands-on procedural experience as a barrier for their fellows to prepare for independent practice; this review suggested simulation training as a possible solution [15]. Therefore, we suggest that PHM fellowship programs may need to partner with other subspecialty fellowship programs, such as critical care, emergency medicine, neonatology, or anesthesiology, to provide their fellows with adequate exposure to neonatal resuscitation, sedation, and point-of-care ultrasound. If these opportunities exist, fellows may need to be encouraged to use their individualized curriculum time to practice these skills, especially if they are pursuing specific jobs that involve these responsibilities. Simulation is another curricular modality that can be used by PHM fellowship programs to teach these skills [16]. For coding and billing, skills that have been highlighted as not well taught during residency [17], PHM fellowships can devote some of their educational curricula to practicing and mastering billing, and identify billing as a specific learning goal when their fellow is on PHM service.

Our results also showed that graduates felt less prepared to navigate the job search and promotion process, and we discovered that many fellowship programs do not include career development within their curricula. These results align with the demographics reported by Rassbach et al, which found more than 86% of respondents were at the assistant professor or instructor level [9]. Additionally, our findings support the results found in a survey study that explored the current PHM workforce dynamics; the authors found that the majority of their respondents, who were all PHM fellowship trained, expected career advancement with earlier promotion due to their fellowship training [18]. Mentors play a critical role in helping new hires understand the promotion process and launching them on the proper trajectory towards promotion [19]. However, in a 2021 survey of pediatric hospitalists, only 57.7% (317/549) reported having a mentor, with some relying on informal peer mentorship [20]. Therefore, we recommend that fellowship programs identify mentors who can train and guide fellows for those aspects of their job that are not always covered in the fellowship, such as searching for a job, negotiation, and promotions. 

Our study also addresses the gap in the literature regarding perceived job satisfaction after training and the challenges new hires face in their first job as an attending, particularly in the fields of pediatrics and specifically pediatric hospital medicine. Our results can also help PHM division leaders when hiring and onboarding new PHM attendings, as they can assist new hires in addressing the training gaps that remain after fellowship. PHM divisions can provide faculty development in areas that fellowships are unable to provide or do not provide enough of. For example, faculty development can focus on billing, leadership skills, preparing for promotion, and scholarship. PHM division leaders can also be intentional about helping their new hires find and identify a mentor (e.g., research mentor or career mentor), especially if the new hire is new to the institution. This is especially important since it has been shown that hospitalists without mentors are less likely to be academically productive [21]. These recommendations may help PHM divisions to ease the transition from fellow to attending and promote scholarship and academic advancement.

Limitations 

Our study has several limitations. While we report on graduates from a single specialty, many of the findings may be generalizable to other hospital-based specialties. Non-response bias may also limit our results, though we tried to make the survey simple and easy to complete within 10 minutes. Similarly, despite the small sample size, we contacted all institutions that offered a PHM fellowship in 2020 and achieved a 50% response rate. We asked participants to recall experiences from the fellowship, so their answers may be affected by recall bias, although participants completed the survey approximately one year after graduation. Lastly, we did not fully account for the potential impact of the COVID-19 pandemic, which may have contributed to unique stressors that the 2020 graduates faced. Future studies should evaluate whether post-pandemic graduates have similar survey responses.

## Conclusions

2020 marked the first year that pediatricians were required to complete a PHM fellowship to be PHM subspecialty board-certified by the American Board of Pediatrics (ABP). Since then, the number of PHM fellowship programs and positions has continued to grow. Our study reveals which domains PHM fellowship graduates felt adequately prepared for as attending physicians, information critical for PHM fellowship program directors to have as they strive to optimize curricular experiences and prepare fellows for successful transition into their professional careers. While this study only assessed PHM fellowships, these results may be useful to other subspecialties as they design their curricula and prepare their fellows for the transition to attending.

## Appendices

Appendix 1. Survey instrument

1. Consider the following factors that were important in choosing your first job after the PHM fellowship. Please identify and rank the five most important factors that you considered. (1 = most important, 2 = second most important, etc.). One selection allowed per column.

**Table 4:** Most important factors in choosing your first job after PHM fellowship Template for recording the responses on what potential factors (e.g., location, salary, division size) are ranked among the top five considerations by participants when selecting their first job after the PHM fellowship PHM: pediatric hospital medicine

	Most important	Second most important	Third most important	Fourth most important	Fifth most important
Location					
Salary					
Familiarity with the institution					
Opportunities for research					
Incentives (i.e., sign on bonus, relocation package, etc.)					
Family					
Work schedule					
Hospital’s mission statement					
Dynamics within/culture of the hospitalist division					
Division Chief					
Department Chair					
Affiliation with medical school/academic faculty title					
Recruited to join/stay where I trained					
Supportive environment for underrepresented minorities (URM)					
Support for junior faculty (i.e., mentorship for academic career, leadership training)					
Size of the division					
Other, please explain					

2. How confident are you that the fellowship prepared you for your first job?

a. Not at all confident

b. Slightly confident

c. Moderately confident

d. Quite confident

e. Extremely confident

3. Thinking back to these topics covered during your fellowship, how would you rate your preparedness?

**Table 5:** Rate your preparedness Template for recording participants’ self-assessed preparedness across multiple aspects of their first attending role after PHM fellowship, using a 5-point Likert scale (1 = not at all prepared, 5 = extremely prepared) PHM: pediatric hospital medicine

	Extremely prepared	Quite prepared	Moderately prepared	Slightly prepared	Not at all prepared
Billing					
Teaching residents					
Teaching medical students					
The promotion process					
Surgical co-management					
Managing complex care patients					
Sedation					
Performing a lumbar puncture					
Point of care ultrasound					
Pediatric resuscitation and stabilization					
Neonatal resuscitation					
Leading family centered rounds					
Quality improvement					
Clinical research					
Medical education research					
Mentoring residents					
Mentoring medical students					
Pain management					
Hospital medicine consults					
Working with non-physician providers (i.e., physician assistant, nurse practitioner)					
Operational leadership (i.e., medical director)					
Publishing scholarly work					
Navigating hospital politics					
Maintaining wellness					
Finding a job after fellowship					

4. How satisfied are you with your fellowship program?

a. Not at all satisfied

b. Slightly satisfied

c. Moderately satisfied

d. Quite satisfied

e. Extremely satisfied

5. How satisfied are you with your primary fellowship director?

a. Not at all satisfied

b. Slightly satisfied

c. Moderately satisfied

d. Quite satisfied

e. Extremely satisfied

6. How helpful was your fellowship director in finding your first job?

a. Not at all helpful

b. Slightly helpful

c. Moderately helpful

d. Quite helpful

e. Extremely helpful

7. What surprised you the most about your first job?

a. Free text

8. Why were you surprised?

a. Free text

9. Have you met with a mentor at your first job?

a. Yes

b. No

10. How satisfied are you with your first job after the fellowship?

a. Not at all satisfied

b. Slightly satisfied

c. Moderately satisfied

d. Quite satisfied

e. Extremely satisfied

11. What was the most significant challenge transitioning from fellow to attending?

a. Free text

12. What barrier(s) did you encounter in finding the right job after the fellowship?

a. Free text

13. What is one thing you wish the fellowship focused on more that would have been beneficial to you as a new hire?

a. Free text

14. Did your fellowship offer a career development curriculum?

a. Yes - at the fellowship program level

b. Yes - at the departmental/institutional level

c. Yes - at both levels

d. No

e. Unsure

Demographics

15. What is your gender identity?

a. Male

b. Female

c. Non-binary

d. Another gender identity

e. Would prefer not to answer

16. What is your race/ethnicity (check as many as apply)?

a. African or Afro-Caribbean

b. Black or African American

c. American Indian or Alaskan Native

d. Native Hawaiian or Other Pacific Islander

e. Filipino

f. South Asian (Indian, Pakistani, Bangladeshi, or other South Asian)

g. East Asian (Chinese, Japanese, Korean, or other East Asian)

h. Asian-American

i. Latinx, or of Spanish origin

j. Middle Eastern or North African

k. Arab American

l. White or European American

m. Multiracial

n. Other, please describe

o. Prefer not to say

17. What is your age?

a. 25 - 30 years

b. 31 - 35 years

c. 36 - 40 years

d. >40 years

18. What was the length of your fellowship?

a. 2 years

b. 3 years

19. What year did you graduate from your fellowship?

a. Free text

**Table 6:** The counts of the most surprising things about the first job This table lists the most commonly reported unexpected aspects of transitioning from fellowship to the first attending role, as reported by respondents in open-ended survey questions. Data were derived from free-text responses and coded by three independent reviewers

Keywords	Exact words from answers	Counts
Different from fellowship	“How different the clinical duties/expectations differed from the PHM department where I trained.”	6
	“We didn't have to be in office on our off service weeks.”	
	“culture of the division…was very different…”	
	“…difference in team dynamics and support for hospitalists…”	
	“…difference in seeing patients …”	
	“Learning the behind-the-scenes of hiring…”	
Difficulty in finding mentorship	“Difficulty in finding mentorship as a junior faculty…”	5
	“Difficulty finding the right mentorship…”	
	“Less formal mentorship”	
	“…difficulty getting a consultant providers help with complex patients…”	
	“1. The lack of mentorship…”	
Difficulty in adapting to the changes	“…it was significantly worse at the new institution…”	3
	“stress of being the final decision maker, decision fatigue from heavy clinical burden. Intricacies of billing.”	
	“…2. Multiple system/infrastructure/organizational issues that had a significantly negative impact…3. …demoralizing lack of resources…”	
Smooth transition	“I ended up in such a supportive environment, and the transition went so smoothly…”	3
	“…pleasantly surprised… It is so easy to either start or get involved in projects at a hospital that is passionate about caring for kids and has a team-based approach”	
	“I love my job far more now that I'm an attending…”	
Increase in workload	“The work schedule being more draining…”	2
	“How much the work load increased…”	
Difficulty in find a job	“…not as easy to find a job…”	2
	“That I would be doing newborn nursery time, was part of what was available at the time.”	
The reality of job not as expected from the interview	“Reality if the job dynamics (patient load, obtaining protected research/teaching time) compared to what was discussed on the interview”	1
Nothing	“nothing”	1

**Table 7:** The counts of the reported reasons for being surprised by the first job This table categorizes participants’ explanations for why certain aspects of their first job were surprising. Data were derived from free-text responses and coded by three independent reviewers

Key information	Exact words from answers	Counts
Differences in institutional culture	“Was not the same institutional culture from where I trained.”	3
	“…I was sheltered by my attendings in training and I wasn't aware of the politics of the hospital”	
	“see above- The extreme difference in team dynamics and support for hospitalists…”	
Less working time during fellowship	“I calculated the hours as not being that much more than what we did during fellowship, but it just takes much more out of you.”	2
	“…also the amount of time spent on resident run service was much less.”	
Expected more preparedness for job search after fellowship	“I went to fellowship after residency thinking that the fellowship would set me up for lots of job opportunities.”	2
	“I felt fellowship training would set me up to be the most competitive but it ultimately depended on the tight market as to what was available”	
Took for granted the mentorship during fellowship	“As a fellow I took for granted the mentorship structure as well as bought out time for mentors to serve in that role for fellows…”	1
N/A	“N/A”	1
Expected less workload at job	“Always thought the attending life would be easier than training.”	1
Never had the similar experience before	“Never been on the other end of hiring people before for full time jobs (not fellowship positions) …”	1
Unable to visit and learn more before onboarding	“Possibly being unable to visit on-site. The job was also in a geographical region far from fellowship training…”	1
Received different message than the reality	“Different message than the reality”	1

**Table 8:** The counts of the reported challenges transitioning from fellow to attending This table summarizes reported challenges experienced during the transition from PHM fellowship to independent attending practice. Responses were drawn from open-ended survey questions and coded by three independent reviewers PHM: pediatric hospital medicine

Key information	Exact words from the answers	Counts
Increased clinical responsibility and less supervision or support as an attending vs. a fellow	“…stress of knowing that I have the liability…”	11
	“Added stress of increased clinical responsibility coupled with less protected time”	
	“Not having a designated backup person to run things by in cases of clinical uncertainty”	
	“…as nobody else is looking over my shoulder”	
	“Confidence in clinical decision making”	
	“Clinical responsibility”	
	“Extreme difference in …and support during nights”	
	“Stress of being the final decision maker”	
	“Taking calls from outside hospitals”	
	“Less support available to discuss clinical/management questions”	
Increased workload	“Increase in clinical work load…”	3
	“The volume of patients to manage.”	
	“Extreme difference in patient load…”	
Difficulty in scholarly work	“Navigating how to get into scholarly work…”	3
	“…starting scholarly work”	
	“…trying to find time to complete projects…”	
Learn a new system	“Learning a new system…”	4
	“…learning a new hospital.”	
	“Learning new institution”	
	“…Navigating a broken system after graduating from a high-functioning, leading, medical care system.”	
Difficulty in getting people recognized	“…still view me as a trainee”	2
	“…people still saw me as a fellow”	
Difficulty in finding mentorship	“Difficulty finding mentorship”	2
	“Finding Mentorship and…”	
Billing	“Probably either billing…”	1
Clinical relationships	“1. Establishing clinical relationships…2. Navigating differences in clinical practice approaches…”	1

**Table 9:** The counts of the reported barriers participants encountered in finding the right job after fellowship This table displays participant-reported obstacles encountered when searching for their first job post-fellowship. Data were coded from open-ended responses into themes by three independent reviewers

Key information	Exact words from the answers	counts
Lack of job availability	“Pandemic...there were hardly any jobs available…”	10 (5 mentioned COVID/pandemic)
	“COVID pandemic and loss of job availability…”	
	“Job availability in the market…”	
	“Not enough jobs available.”	
	“Job availability during pandemic”	
	“limited open positions.”	
	“The job market was quite limited…”	
	“Job availability (especially when COVID hit)”	
	“Lack of jobs during the pandemic!”	
	“Availability, finding the right fit”	
Balancing various factors in choosing the job	“Balancing work-life balance, balancing an institution's reputation vs available opportunities, location”	5
	“Considering both IM and peds time as a med-peds trained PHM fellow”	
	“Deciding which important factors to prioritize…”	
	“Desire to be in a specific location limited opportunities”	
	“…balanced clinical duties with appropriate opportunities for advancement/projects.”	
None	“No barriers”	3
	“None.”	
	“None”	
Salary	“Negotiating salary…”	2
	“Salaries not as competitive as I thought”	
Arrange time for different positions/offers	“…Deadline to respond to job offers Ensuring significant other also had job opportunities in the same location”	2
	“…timing was difficult (eg one job wanted a reply before another job gave me a response)”	
“Inside track”	“Some institutions, preferred candidates that graduated from their program or whom they already knew instead of the most qualified candidate.”	1
